# Rational construction of PCL-PEG/CS/AST nanofiber for bone repair and regeneration

**DOI:** 10.3389/fbioe.2024.1515043

**Published:** 2025-01-10

**Authors:** Zhengyu Cao, Hongwu Zhuo, Wendong Zhu, Xiangfang Peng, Jian Li

**Affiliations:** ^1^ Department of Sports Medicine, The Second Affiliated Hospital of Fujian University of Traditional Chinese Medicine (FJTCM), Fuzhou, China; ^2^ Key Laboratory of Polymer Materials and Products of Universities in Fujian, Department of Materials Science and Engineering, Fujian University of Technology, Fuzhou, Fujian, China

**Keywords:** electrospinning, nanofiber, humerus, bone, tuberosity

## Abstract

Humerus greater tuberosity (HGT) avulsion fracture is one of the most common types of proximal humerus fractures. The presence of motion and gap lead to the failure of implants, due to the force pulling from the supraspinatus. In this work, electrospinning technology was applied to fabricate PCL-PEG/CS/AST nanofiber with superior biocompatibility and mechanical property. Furthermore, PCL-PEG/CS/AST nanofiber could promote proliferation and osteogenic differentiation of bone mesenchymal stem cells (BMSCs) *in vitro*. We believe that this work indicates a promising way to promote the union of HGT avulsion fractures by using PCL-PEG/CS/AST nanofiber.

## 1 Introduction

Bone repair and bone regeneration are crucial task in clinical treatment. The healing of the humerus greater tubercle (HGT) is highly important. As the main attachment point of the rotator cuff, humerus greater tubercle (HGT) plays an important role in maintaining the function of shoulder joint abduction and rotation (Lacheta et al., 2023; Bekmezci et al., 2024). HGT avulsion fractures are among the most common types of proximal humerus fractures, especially in the osteoporosis population, accounting for approximately 20% of proximal humeral fractures (Handoll et al., 2022; Kim et al., 2024b). Arthroscopic suture anchor and locking plate fixation are common methods in the clinical treatment of HGT avulsion fractures, however, surgical treatments still have high failure rates, and adverse events, such as internal fixation failure and fracture displacement often occur (Makaram et al., 2023; Kim et al., 2024b; Tao et al., 2024). Previous studies reported that the suture anchor technique requires adequate bone mineral density to hold the anchor and that the anchors are easily pulled out in patients with severe osteoporosis around the proximal humerus (Lee S. et al., 2021; Kim et al., 2023). In addition, many studies founded that the presence of motion and gaps due to pulling from the supraspinatus, which is known to delay the union of fractures, eventually leaded to failure of internal fixation (Zeng et al., 2021; Handoll et al., 2022). Thus, better approaches for promoting HGT avulsion fracture healing, which are essential for the recovery of shoulder function, are needed for elderly and osteoporotic population.

In recent years, many types of material have been developed and utilized in bone repair and bone regeneration. Among them, nanofiber is an ideal scaffold for therapeutic medicines, with the features of huge aspect ratio, specific surface area, flexibility, and mechanical strength (Zhu et al., 2021; Cheng et al., 2021; Cheng et al., 2022b; Cheng et al., 2022a). Many polymer nanofibers with excellent biocompatibility have shown outstanding results for prosthetics, including polylactic acid (PLA), polycaprolactone (PCL), polyethylene glycol (PEG) and numerous biomolecules. For example, Kim et al. (2024a) utilized the oxygen plasm to treat PCL nanofibrous scaffold, aiming to improve the hydrophilicity and protein adsorption properties. As the results illustrated the treated PCL nanofiber showed dramatically improved new bone formation behavior (Kim et al., 2024a). As a one of the natural polysaccharides, chitosan owns an unique chemical structure, which attracted enormous interest in controlled drug delivery, gene delivery, cell culture, and tissue engineering. After assisting by bioactivated magnesium-doped hydroxyapatite, electrospun chitosan nanofiber scaffolds simultaneously displayed the great bone mineralization ability (Sedghi et al., 2020). To further improve the effects of bone repair and regeneration, various bioactivated materials are added into the scaffold, including drugs, ceramics, metal-organic frameworks, semiconductor materials (Wang et al., 2019; Liu et al., 2022; Sun et al., 2022; Fan et al., 2024; Makurat-Kasprolewicz et al., 2024). Alendronate sodium ([(4-amino-1-hydroxybutylidene)-bisphosphonate] trihydrate) have the capability inhibit the bone remodeling activity and resorption by interacting with bone matrix to treat osteoporosis and other osteolytic bone diseases (Akyol et al., 2015; Doca et al., 2016). He et al. (2018) chosen Alendronate Sodium modified the collagen type I for bone regeneration, because the Phosphorylated materials could provide the beneficial environment of extracellular matrix. Compared with the treatment of 4 weeks, the new bone formation is apparent after treatment of 8 weeks (He et al., 2018).

In this work, PCL-PEG/CS/AST nanofiber was prepared via electrospinning technology. The fibrous morphology of PCL-PEG/CS/AST nanofiber is beneficial for cell adhesion and proliferation, exhibiting superior biocompatibility. Furthermore, an adhesive-nanofiber membrane based approach was proposed to promote HGT avulsion fracture healing. We hypothesized that PCL-PEG/CS/AST nanofiber membrane could enhance HGT avulsion fracture healing by promoting osteogenesis and reducing the failure rate after surgery. We believe this work provides an universal and simple approach for bone repair and bone regeneration with potential insight.

## 2 Materials and methods

### 2.1 Materials and reagent

Polycaprolactone-polyethylene glycol copolymer (PCL-PEG) was synthesized from Ruijiu Technology Co., Ltd. Chitosan (CS, deacetylation degree ≥95%, viscosity: 100–200 Mpa/s), sodium alendronate (AST), hexafluoroisopropyl alcohol (HFIP) were purchased from Shanghai Macklin Biochemical Technology Co., Ltd. Alpha-modified minimal essential medium (α-MEM), penicillin–streptomycin (P/S) and fetal bovine serum (FBS) were purchased from Thermo Fisher Scientific (Scoresby, Vic., Australia). The Cell Counting Kit-8 (CCK-8) was purchased from Bioscience (Shanghai, China). Alkaline Phosphatase (ALP) Assay Kit was purchased from Beyotime (Shanghai, China). Live and Dead™ Viability Assay Kit and rhodamine-conjugated phalloidin were purchased from US Everbright Inc (Suzhou, China). The universal RNA extraction kits and Evo M-MLV RT kits were purchased from Accurate Biotechnology Co., Ltd. (Hunan, China). The primers that used in this study were purchased from Sangon (Shanghai, China). All reagents were used directly without pretreatment.

### 2.2 Characterizations

The morphologies of PCL-PEG/CS/AST nanofiber membrane was achieved by field emission scanning electron microscope (FE-SEM, Regulus 8100). The chemical structure of PCL-PEG/CS/AST nanofiber membrane was analyzed by Fourier Transform infrared spectroscopy (FTIR, Nicolet IS350). The valence information and surface composition of PCL-PEG/CS/AST nanofiber membranes were analyzed by X-ray photoelectron spectroscopy (XPS, ESCALAB QXi).

### 2.3 Preparation of PCL-PEG/CS/AST nanofiber membrane

0.2 g of PCL-PEG was added into 4.2 g of HFIP and kept stirring to forming a homogeneous solution. Then, 0.04 g of CS and 0.012 g AST were injected to the above-mentioned solution. Subsquently, the precursor solution was loaded into a syringe with a single stainless steel nozzle for electrospinning. The voltage supply was maintained at 15 kV. Then, the PCL-PEG/CS/AST nanofiber membrane were prepared.

### 2.4 BMSCs cultivation, BMSCs viability assay and live-dead cell staining

BMSCs were purchased from Shanghai Zhong Qiao Xin Zhou Biotechnology Co., Ltd. BMSCs were cultured in α-MEM complete culture medium for 72 h and the culture medium was changed every 48 h until BMSCs reached 80% confluence. BMSCs were seeded on the surfaces of PCL/PEG, PCL-PEG/ALN, PCL-PEG/CTS and PCL-PEG/ALN/CTS with the density of 5 × 10^4^/well in a 12-well plate, respectively (3 duplicate wells for each sample). The viability of the BMSCs on the surfaces of the four samples was detected on the third and seventh days, respectively, which is according to a related published article. (Zhang et al., 2022). Meanwhile, BMSCs on the samples were fixed and stained according to the live-dead cell staining kit manufacturer’s protocol at day third and seventh, respectively. Image J software was used to count the living and dead cells on the surface of nanofiber membranes, respectively.

### 2.5 Osteogenic differentiation *in vitro*

For quantitative real-time polymerase chain reaction (qRT-PCR) and ALP activity, BMSCs were seeded on each sample as described above and α-MEM complete culture medium was changed to the osteoblast inducing conditional medium when BMSCs reached 100% confluence. ALP activity of BMSCs was detected according to the manufacturer’s protocol after 14 days of osteogenic induction. The absorbance was detected at 405 nm with a microplate reader. The total RNA of BMSCs on the surface of bionanofiber membranes was extracted after 14 days of osteogenic induction via a Universal RNA Extraction Kit, respectively. Then, the total RNA was reverse transcribed into cDNA with an Evo M-MLV RT kit and the data were analyzed by the 2^-△△CT^ method. The sequences of the primers of osteogenic genes were shown in Table 1.

**TABLE 1:** The sequences of the primers of osteogenic genes.

Gene	Forward primer	Reverse primer
ALP	CCA​CTA​TGT​CTG​GAA​CCG​CA	GGA​GAG​CGA​AGG​GTC​AGT​C
Osteocalcin	GAC​CAT​CTT​TCT​GCT​CAC​TCT​GC	ACC​TTA​TTG​CCC​TCC​TGC​TTG
GAPDH	AGGAGAGTTTCCTCGTCC	TGA​GGT​CAA​TGA​AAG​GGG​TCG

### 2.6 Statistical analysis

In this study, all experiments were repeated at least 3 times. The results were presented as mean ± standard deviation (SD) if the data obeyed normal distribution. GraphPad Prism (version 7, GraphPad Software, San Diego, United States) was used for statistical analysis and statistical graphs. One-way ANOVA with Tukey’s post hoc test was used to determine the significant differences among several groups. P values <0.05 were considered to indicate statistically significant differences.

## 3 Results and discussion

**FIGURE 1:**
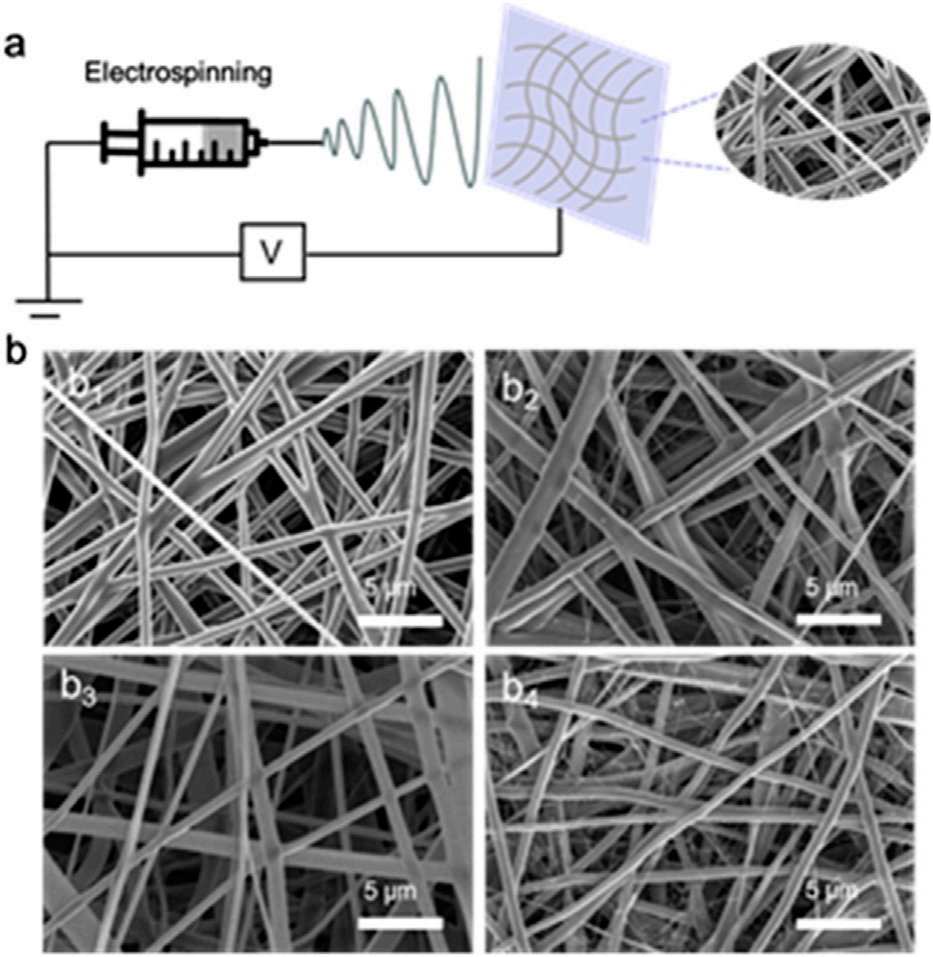
**(A)** Preparation process diagram of PCL-PEG/CS/AST nanofiber; **(B**_**1**_ **)** SEM image of the prepared PCL-PEG nanofiber membrane; **(B**_**2**_ **)** SEM image of the prepared PCL-PEG/CS nanofiber membrane; **(B**_**3**_ **)** SEM image of the prepared PCL-PEG/AST nanofiber membrane; **(B**_**4**_ **)** SEM image of the prepared PCL-PEG/CS/AST nanofiber membrane.

The typical synthesis process of PCL-PEG/CS/AST nanofiber is shown in Figure 1A. The PCL-PEG/CS/AS nanofiber membrane was prepared by electrospinning technology. As shown in Figure 1B_1_, PCL-PEG nanofiber exhibit smooth surface and fibrous morphology, which is suitable for substrate (Figure 1B_1_). Figure 1B_2_ displays the SEM images of PCL-PEG/CS nanofiber, flattening and overlap of nanofiber can be observed, which could be assigned to the presence of chitosan. The SEM images of is PCL-PEG/AST nanofiber reveals the smooth fibrous morphology (Figure 1B_3_). As illustrated in Figure 1B_4_, the morphology of the PCL-PEG/CS/AST nanofiber fiber does not change, indicating that the addition of CS and AST displayed no effect on the morphology of the nanofiber.

**FIGURE 2:**
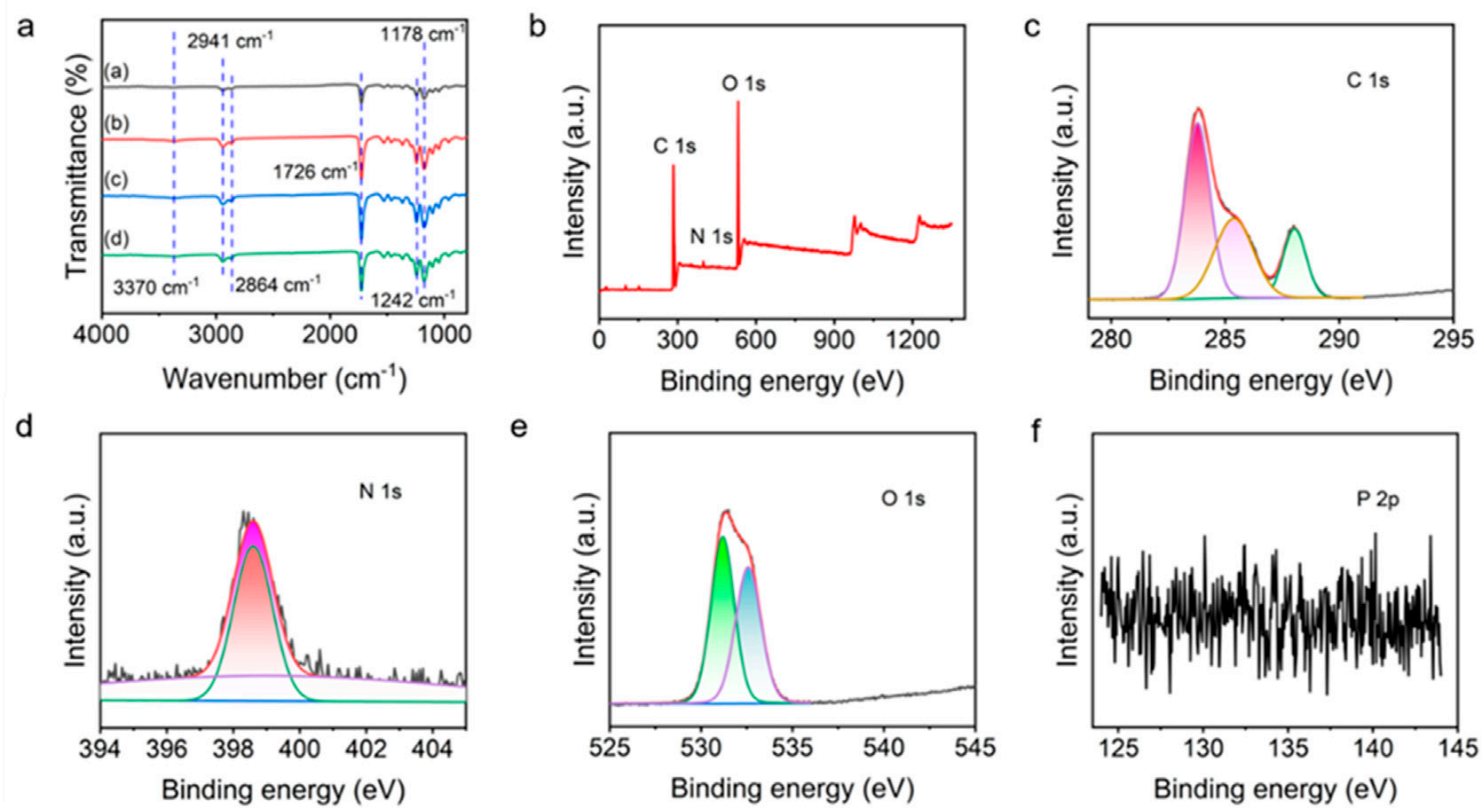
**(A)** FTIR spectra of **(A)** PCL-PEG/CS/AST nanofibers, **(B)** PCL-PEG/AST nanofibers, **(C)** PCL-PEG/CS nanofibers and **(D)** PCL-PEG nanofibers. **(B)**. Full XPS survey of prepared PCL-PEG/CS/AST nanofibers. The fine XPS spectra of the PCL-PEG/CS/AST nanofibers, **(C)** C1s, (**D)** N1s, **(E)** O 1s, and **(F)** P 2p regions.

The chemical structure and different types of chemical bonds of composites were investigated by FTIR spectroscopy. As shown in Figure 2A, the characteristic peaks at 2,941 cm^−1^ and 2,864 cm^−1^ correspond to the C-H asymmetric and symmetric stretching of the carbonyl group, the characteristic peak at 1726 cm^−1^ is attributed to the stretching movement of C=O, and the characteristic peaks near 1,242 cm^−1^ and 1,178 cm^−1^ prove the formation of COC asymmetric and symmetric stretching, respectively (Deng et al., 2021). Hydrogen bond interactions may occur in PCL-PEG copolymers (Yu et al., 2014), and the peak at 3,370 cm^−1^ confirmed the presence of O-H in the mixture. After the introduction of CS and AST, it is found that the spectrum changes little. However, in the nanofibers containing CS and AST, the peak strength near 1726 cm^−1^ is reduced, illustrating the difference in the amount of C=O in the mixture (Deng et al., 2021), which also indicates that CS and AST are successfully imported. No differences among PCL-PEG (d) and PCL-PEG/CS/AST (a), PCL-PEG/AST (b), and PCL-PEG/CS (c) blends were detected in Figure 2A, which may be due to the low contents of CS and AST in polymer matrix. All FTIR test results prove the successful preparation of PCL-PEG/CS/AST nanofiber membrane.

XPS was applied to further characterize the surface elemental states of PCL-PEG/CS/AST nanofiber (Figures 2B–F). The presence of C, N, and O elements can be clearly calrified in the full spectrum of PCL-PEG/CS/AST. In the fine spectrum of C 1s, it is located at 284.0, 285.4, 288.0 eV, which is corresponded to C-C/C=C, C-C/C-H, hydrocarbons and C=O (Al-Ani et al., 2018; Manakhov et al., 2019; Liu et al., 2020; Yingjun et al., 2023). The fine spectrum d of N 1s can be deconvolved into 398.6 and 399.2 eV, which could be assigned to the presence of N-C and NH_2_ (Wang and Liu, 2013; Petrović et al., 2022). In the fine spectrum of O 1s, the peaks displayed at 531.2 and 532.6 eV, which is related to C=O and N-C=O in the N-acetylated-glucosamine units (Wang and Liu, 2013; Yingjun et al., 2023). In the full spectrum of PCL-PEG/CS/AST nanofiber and the fine spectrum of P 2p, there are no obvious characteristic peaks related to P was found, which also confirm the trace content of AST in PCL-PEG/CS/AST nanofiber. All XPS test results indicate the successful preparation of PCL-PEG/CS/AST nanofiber membrane.

**FIGURE 3:**
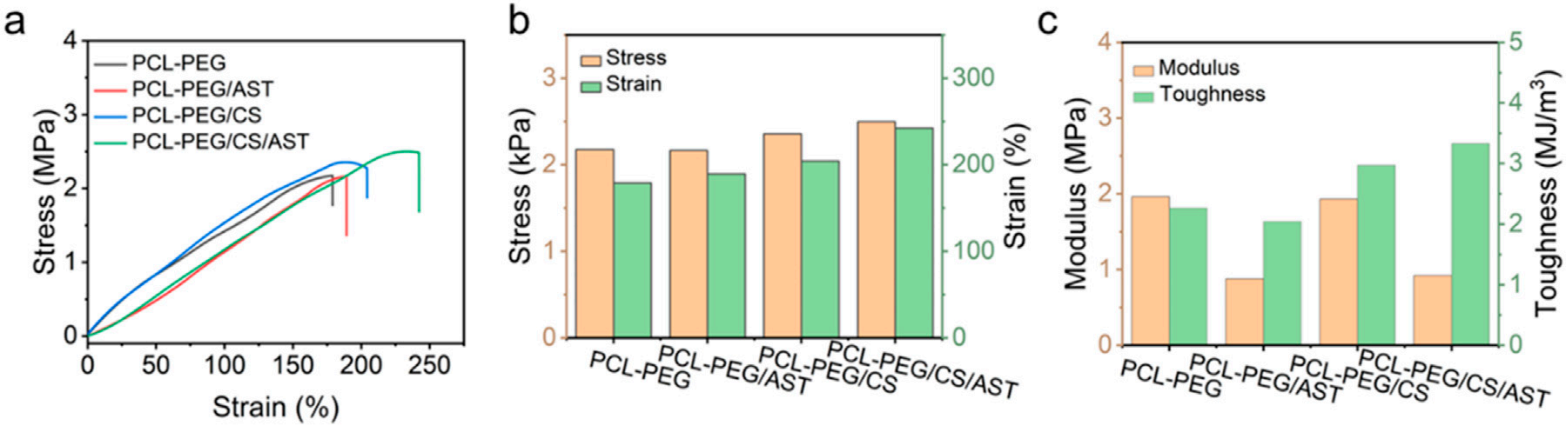
**(A)** Stress–strain curves, histograms of **(B)** stress, strain, **(C)** modulus and toughness of PCL-PEG/CS/AST, PCL-PEG/CS, PCL-PEG/AST and PCL-PEG nanofiber membrance.

The mechanical property of PCL-PEG/CS/AST nanofiber membrane was also clarified. As shown in Figure 3, the stress, elongation at break, elastic modulus, and toughness of PCL-PEG/CS/AST nanofiber membrane reach 2.50 MPa, 2,242.33%, 0.92 MPa, and 3.33 kJ/m^3^, respectively, proving its favorable mechanical performance. Furthermore, the long-term stability of PCL-PEG/CS/AST nanofiber membrane can be predicted. According to the previous work, Polymer dispersity index (PDI), Zeta potential was applied to verify the long-term stability with satisfactory result in 25°C for 14 days (Wang et al., 2021). Chitosan exhibited the superior stability in varied conditions (high-temperature, high-salt, and long-term storage at 4°C) (Li et al., 2024). Thus, we believe PCL-PEG/CS/AST nanofibers also present the favorable long-term stability.

### 3.1 Cell viability

**FIGURE 4:**
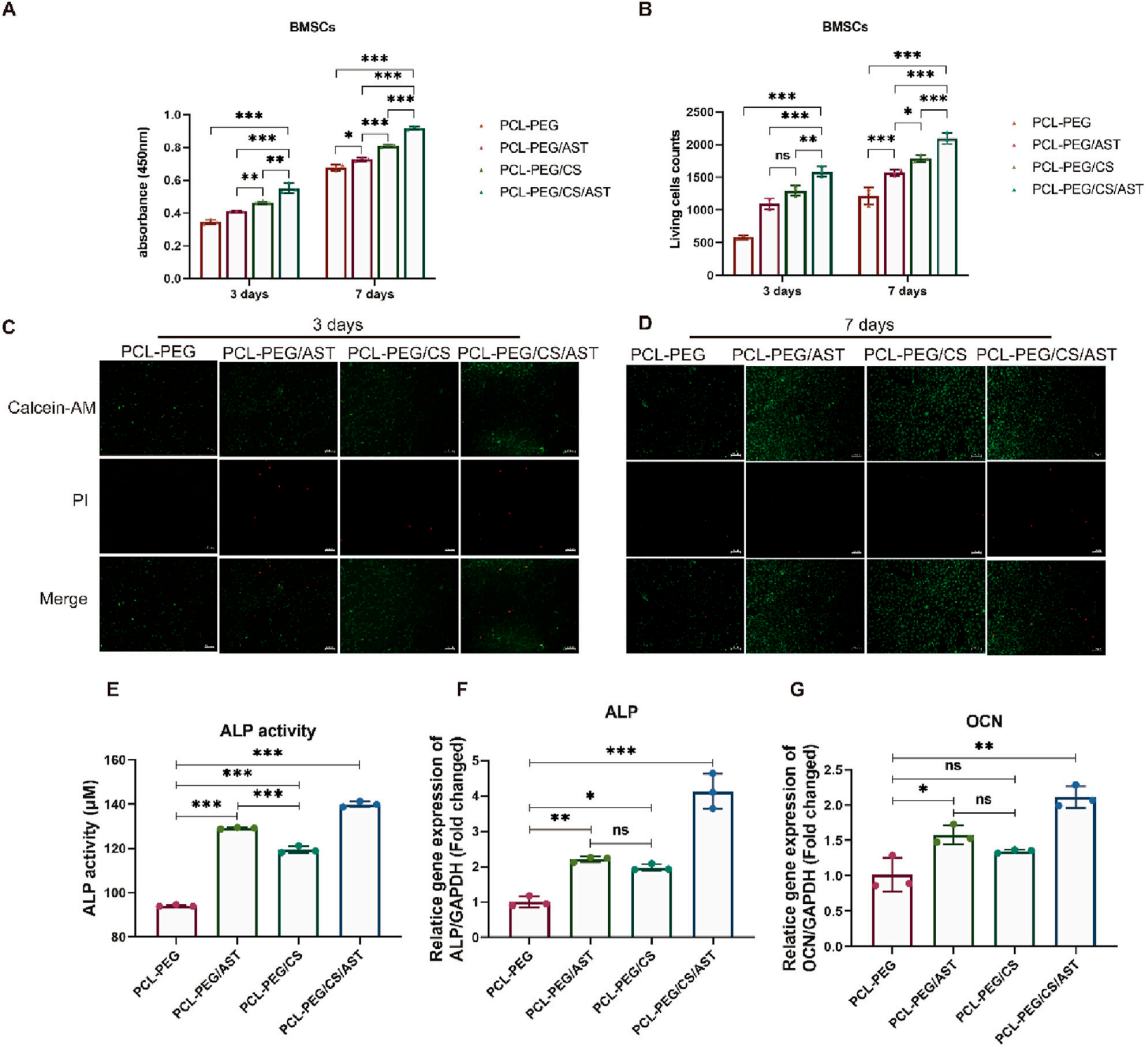
**(A)** The proliferation rate of BMSCs on the surface of each biofilm after inoculation for 3 and 7 days. **(B)** The counts of Living BMSCs on the surface of each biofilm after inoculation for 3 and 7 days. **(C)** The images of living BMSCs and dead BMSCs on the surface of each biofilm after inoculation for 3 days. **(D)** The images of living BMSCs and dead BMSCs on the surface of each biofilm after inoculation for 7 days. **(E)** ALP activity of BMSCs on each biofilm after 14 days of osteogenic induction. **(F)** ALP expression of BMSCs on each biofilm after osteogenic induction for 14 days. **(G)** OCN expression of BMSCs on each biofilm after osteogenic induction for 14 days. *P < 0.05; **P < 0.01; ***P < 0.005; ns, no significant.

In Figure 4A, the proliferation rate of BMSCs on the surface of PCL-PEG/CS nanofiber membrance (0.46 ± 0.01) and PCL-PEG/CS/AST nanofiber membrane (0.55 ± 0.03) was significantly promoted compared with that of the former 2 groups (0.35 ± 0.01, 0.41 ± 0.01, respectively) after inoculation for 3 days (P < 0.05). The result of cell viability test after inoculation for 7 days revealed a similar trend that the proliferation rate of BMSCs on the surface of PCL-PEG/CS nanofiber membrance (0.81 ± 0.01) and PCL-PEG/CS/AST nanofiber membrance (0.92 ± 0.01) was significantly promoted compared with that of the former 2 groups (0.68 ± 0.02, 0.73 ± 0.01, respectively).

In Figures 4B–D, Live BMSCs were dyed green with calcein-AM, dead BMSCs were dyed red with propidium iodide, and few dead BMSCs were observed under the fluorescence microscope in all four groups, the result of live-dead cell staining showed the same trend, the number of living cells (1,589.00 ± 83.50) was the highest on the surface of PCL-PEG/CS/AST nanofiber membrance. The number of living cells on the surface of PCL-PEG/CS nanofiber membrance (1,297.33 ± 78.40) was significantly greater than the former 2 groups (597.33 ± 30.37, 1,098.00 ± 86.09, respectively) (P < 0.05).

Similarly, Cai et al. has constructed a kind of chitosan derivative scaffold, which could promote proliferation and osteogenic differentiation of MSCs via reducing intracellular reactive oxygen species (ROS) (Wang et al., 2020). Another related article has found that chitosan could promote proliferation of tonsil-derived mesenchymal stem cells (TMSCs) via up-regulating cyclin D1 in the G1 phase of the cell cycle (Lee K. E. et al., 2021). In addition, Martín-López et al. reported that different concentration of chitosan could affect the polymer surface topography, which has a direct effect on the growth of cell behavior (Martín-López et al., 2013).

### 3.2 Osteogenic differentiation of BMSCs *in vitro*

Alkaline phosphatase (ALP) is a kind of exoenzyme of osteoblast, and its expression activity is a very obvious characteristic of osteoblast differentiation and maturation. As shown in Figure 4E, BMSCs on the surface of PCL-PEG/CS/AST nanofiber membrane had a significantly higher level of ALP activity (139.95 ± 1.27) than other 3 groups (94.11 ± 0.48, 129.20 ± 0.33 and 119.47 ± 1.57, respectively) after 14 days of osteogenic induction (P < 0.05). As shown in Figures 3F, G, the expression level of osteogenic genes in BMSCs on four kinds of nanofiber membrane, including ALP and Osteocalcin (OCN) were detected via qRT‒PCR analysis. Generally, Osteocalcin (OCN) is specifically expressed in osteoblasts and is the most abundant non-collagenous protein in bone, which possess the function of regulating hormone of bone metabolism. The result of ALP gene expression in BMSCs on the surface of PCL-PEG/CS/AST nanofiber membrane (4.13 ± 0.50) had the highest level when compared to the former 3 groups (1.01 ± 0.15, 2.21 ± 0.08 and 1.98 ± 0.09 respectively) (P < 0.05), which showed a similar trend as the result of ALP activity. In addition, the expression level of OCN gene in BMSCs on the surface of PCL-PEG/AST (1.58 ± 0.13) and PCL-PEG/CS/AST nanofiber membrane (2.12 ± 0.15) was significantly higher than those on the surface of PCL-PEG and PCL-PEG/CS nanofiber membrane (1.02 ± 0.24, 1.35 ± 0.02 respectively) (P < 0.05). Chen et al. obtained similar results; they constructed a kind of ALN-loaded hydrogel scaffold and found that the sustained AST release could indeed promote the expression levels of osteogenic-related genes in BMSCs (Tang et al., 2022). Shi et al. had verified that AST served as an optimal osteo-inductive factor to promote osteogenesis within a certain concentration range *in vitro* (Shi et al., 2009; Chang et al., 2022). In addition, Yoon Shin Park et al. reported that chitosan could enhanced the ability of TMSCs to osteoblasts via enhancing its metabolic rate (Lee K. E. et al., 2021), however, significant upregulation of osteogenic genes may cause several side-effects. One notable consequence is the potential for abnormal hyperplasia of HGT, which is one of the common pathogenies of shoulder impingement syndrome in clinic. What’s more, altered bone metabolism, due to osteogenic gene overexpression, may disrupt the delicate balance between bone resorption and formation, which may cause ossification in surrounding soft tissue, such as supraspinatus, infraspinatus and so on.

## 4 Conclusion

In this work, PCL-PEG/CS/AST nanofiber was fabricated via electrospinning technology, exhibiting fibrous morphology, superior biocompatibility and mechanical performance. Furthermore, PCL-PEG/CS/AST nanofiber could promote proliferation and osteogenic differentiation of bone mesenchymal stem cells (BMSCs) *in vitro*. This work provided an innovative way for promoting the union of HGT avulsion fracture with a promising vision of protecting public health.

## Data Availability

The raw data supporting the conclusions of this article will be made available by the authors, without undue reservation.
